# Effect of High-Pressure Treatment on Catalytic and Physicochemical Properties of Pepsin

**DOI:** 10.3390/molecules22101659

**Published:** 2017-10-11

**Authors:** Jianan Wang, Tenghui Bai, Yaping Ma, Hanjun Ma

**Affiliations:** School of Food Science, Henan Institute of Science and Technology, Xinxiang 453003, China; xxjnwang@163.com (J.W.); th2012ky@163.com (T.B.); 13781936901@163.com (Y.M.)

**Keywords:** high-pressure treatment, pepsin, enzymatic hydrolysis, physicochemical properties

## Abstract

For a long time, high-pressure treatment has been used to destroy the compact structures of natural proteins in order to promote subsequent enzymatic hydrolysis. However, there are few reports evaluating the feasibility of directly improving the catalytic capability of proteases by using high-pressure treatments. In this study, the effects of high-pressure treatment on the catalytic capacity and structure of pepsin were investigated, and the relationship between its catalytic properties and changes in its physicochemical properties was explored. It was found that high-pressure treatment could lead to changes of the sulfhydryl group/disulfide bond content, hydrophobicity, hydrodynamic radius, intrinsic viscosity, and subunit composition of pepsin, and the conformational change of pepsin resulted in improvement to its enzymatic activity and hydrolysis efficiency, which had an obvious relationship with the high-pressure treatment conditions.

## 1. Introduction

Proteins and their peptide constituents are important nutrients with key health functions. Numerous studies have shown that the intake of proteins or amino acids alone, even if the amino acid composition is balanced, is not as beneficial as the intake of the peptides derived from enzymatic hydrolysis, which suggests that enzymatically hydrolyzed proteins not only provide amino acid nutrients for the body, but also produce a variety of functional peptides that play a vital role in the health of the body [1,2]. Enzymatic hydrolysis of protein refers to the process by which proteins are degraded into peptides or amino acids with lower molecular weights [3]. Enzymatic hydrolysis is one of the most effective ways to transform proteins, diversify protein functions, and enhance the value of proteins. Because the process is mild, efficient, safe, and easy to control, enzymatic hydrolysis has become one of the most promising directions for development in the global protein processing field [4].

Currently, the bottlenecks and challenges in the enzymatic protein hydrolysis process, including low efficiency and incomplete protein hydrolysis, have greatly restricted the application of enzymatic hydrolysis technology in protein processing. To improve the efficiency of protein enzymatic hydrolysis, some denaturation treatments (e.g., heat denaturation) are usually carried out before hydrolysis, since changing the spatial structure of a protein makes it easier to interact with the proteases, and thereby improves the efficiency and degree of enzymatic hydrolysis [5]. However, these harsh treatments can reduce the biological activity and storage properties of the obtained enzymatic hydrolysates. High-pressure technology is an emerging non-thermal food processing technique that can extend the storage life of products, increase food safety, and improve tissue structure while maintaining the nutritional value and flavor. In particular, it is also an effective technique for changing protein properties [6,7]. Belloque et al. found that the high-pressure treatment could affect the non-covalent hydrogen bonds, ionic bonds, and hydrophobic bonds of the proteins, which could expose more binding sites to prompt the enzymatic hydrolysis [8]. 

However, most studies performed to date on the effects of high-pressure treatment on enzymatic hydrolysis have focused on the substrate (proteins), with the mechanisms underlying the effects of high-pressure treatment on the catalytic and physicochemical properties of the proteases being rarely reported. Pepsin is an important by-product of the meat production process, and is widely used for the enzymatic hydrolysis of protein. In this study, high pressure-treated pepsin was used to hydrolyze casein, and the effects of high-pressure treatment on enzyme activity, degree of hydrolysis, and molecular weight distribution of casein hydrolysate were measured. The effects of high-pressure treatment on the physicochemical properties of pepsin were also investigated.

## 2. Results and Discussion

### 2.1. Effect of High-Pressure Treatment on Enzyme Activity and Degree of Hydrolysis

**Figure 1:**
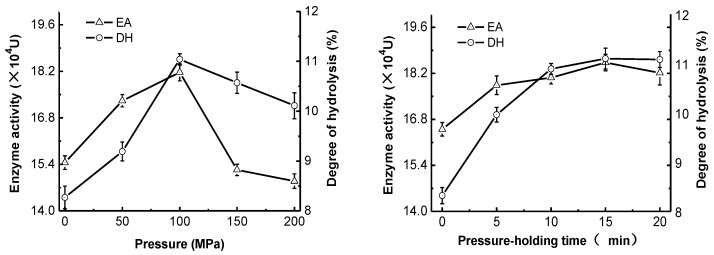
Effect of high-pressure treatment on enzyme activity (EA) and the degree of hydrolysis (DH).

The changes in pepsin activity and degree of casein hydrolysis at different pressures and pressure holding times are shown in Figure 1. Compared with non-pressure-treated samples, the high-pressure treatment with <100 MPa significantly enhanced the pepsin activity (*p* < 0.05) and dramatically enhanced the degree of casein hydrolysis (*p* < 0.05). The maximum enzyme activity (18.17 × 10^4^ U) and the highest degree of hydrolysis (11.04%) occurred at a pressure of 100 MPa, and then declined rapidly with increasing pressure. Under the optimum pressure, the increase of the pressure holding time improved pepsin activity and casein hydrolysis significantly (*p* < 0.05), but no significant changes occurred after 15 min. Therefore, the high-pressure treatment at 100 MPa for 15 min was able to significantly improve the activity and ability of pepsin to hydrolyze casein. Specifically, the enzyme activity and degree of hydrolysis were enhanced from 16.50 × 10^4^ U and 8.32% (blank) to 18.53 × 10^4^ U and 11.1%, respectively, which represented increases of 12.30% and 33.41%, respectively. This suggested that different pressure treatments had changed the enzyme structure and properties to varying degrees, such that enzyme activities and ability to hydrolyze casein had also changed. High-pressure treatment improved the degree of hydrolysis more significantly than enzyme activity. This could be because the structural change induced by high-pressure treatment exposed more enzyme binding sites, which increased the interaction between the enzyme and its substrate.

### 2.2. Effect of High-Pressure Treatment on Molecular Weight Distribution of Casein Hydrolysate

The molecular weight distribution of protein hydrolysate is one of the most important factors to determine its functionality. In this study, high-performance size exclusion chromatography (HPSEC) was used to measure the molecular weight distribution of the casein hydrolysates. The effect of pressure and pressure-holding time on molecular weight distribution of casein hydrolysate is shown in Table 1 and Table 2. The molecular weights of ~83% the peptides in the casein hydrolysates were <2000 u, suggesting that the enzyme had high proteolytic activity, resulting in a high number of short peptides. High-pressure treatment decreased the number of peptides with molecular weights >3000 u and 3000–2000 u, and increased the number of peptides with molecular weights <500 u and 2000–1000 u, which suggested that high-pressure treatment of pepsin could favor the breakdown of high molecular weight peptides into low-molecular-weight peptides. However, there were significant changes in the proportion of peptides with different molecular weights among the samples treated with different pressures (50, 100, 150 and 200 MPa) and pressure holding times (5, 10, 15 and 20 min). High-pressure treatment changed certain enzyme active sites, exposed more cleavage sites, accelerated enzymatic hydrolysis, and significantly increased the amount of each peptide fragment in the hydrolysates (*p* < 0.05). However, the effects of all high-pressure treatment conditions (50–200 MPa for 5–20 min) on the proportion of peptides with different molecular weights tended to be similar during the 6-h proteolytic process. Eisenmenger and Reyes-De-Corcuera [9] found that the effects of high pressure on the enzymatic properties of a protease were complex, which was consistent with our results. The underlying causes may be (1) the direct impact of high pressure on the three-dimensional spatial structure of the enzyme; (2) changes in the mechanism of enzymatic hydrolysis due to pressure (such as a change in the rate-limiting step); (3) high pressure affecting the spatial structure of the enzyme, or changing the rate-limiting steps, by changing the physical properties (such as pH, density, viscosity, or phase) of the substrate or solvent.

### 2.3. Effect of High-Pressure Treatment on the Sulfhydryl Group and Disulfide Bond Content of Pepsin

**Figure 2:**
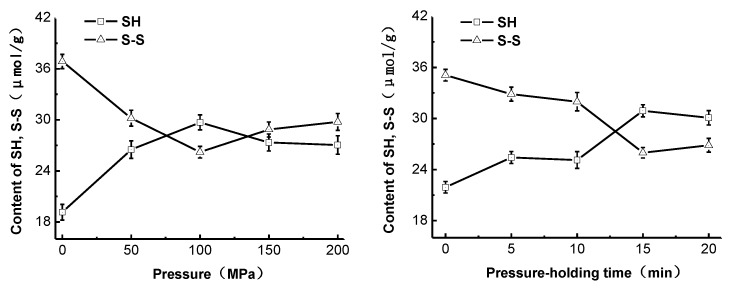
Effect of high-pressure treatment on the sulfhydryl (SH) and disulfide bond (S-S) content of pepsin.

Disulfide bonds are the covalent bonds that are necessary for maintaining the spatial structure and biological activity of proteins. Specific treatments lead to the occurrence of mutual conversion of protein disulfide bonds (S-S) and thiol groups (SH), which allows the protein conformation to become loose and degenerated [10]. In this study, the changes in the SH and S-S contents of pepsin under different high-pressure treatment conditions are shown in Figure 2. It was found that increases in pressure up to 100 MPa resulted in a significant increase in the SH content (*p* < 0.05) and a significant decrease in S-S contents (*p* < 0.05). At pressures higher than 100 MPa, the SH and S-S content were decreased and increased, respectively. Extended pressure holding times below a pressure of 100 MPa increased the SH content and decreased the S-S content significantly (*p* < 0.05), and stabilized after the pressure was held for 15 min. Therefore, the change in the SH content after high-pressure treatment was consistent with the change in degree of hydrolysis, but was the opposite of the change in the S-S content. A possible reason for this was that treatment with 100 MPa for 15 min was able to destroy the spatial structure of pepsin and expand the peptide chains, resulting in the cleavage of hydrophobic and ionic bonds and exposure of SH inside the protein structure to the protein surface, thereby increasing the sulfhydryl group content. The oxidation of thiol groups and the cleavage of disulfide bonds in the protein structure is able to change the SH content, which can reflect the degree of protease denaturation [11]. The reason for the decrease in the SH content and increase in the S-S content under even higher pressures could be that the excessive exposure of SH and the compressed protein structure induced by excessively high pressures could result in the oxidation of some thiol groups into disulfide bonds.

### 2.4. Effect of High-Pressure Treatment on Surface Hydrophobicity, Intrinsic Viscosity and Hydrodynamic Radius of Pepsin

**Figure 3:**
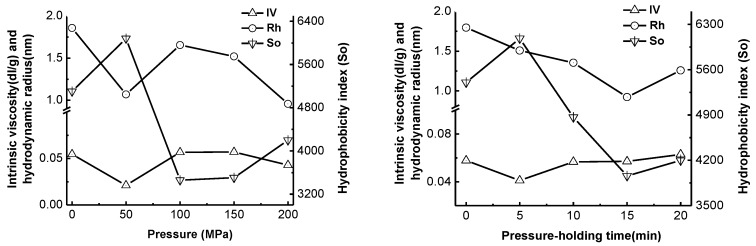
Effect of high-pressure treatment on the surface hydrophobicity (So), intrinsic viscosity (IV), and hydrodynamic radius (Rh) of pepsin.

The surface hydrophobicity (So), intrinsic viscosity (IV) and hydrodynamic radius (Rh) are important indicators of the structures of proteins, and can be used to evaluate the effect of processing on the denaturation and aggregation of proteins [12]. As shown in Figure 3, the increase in pressure up to 50 MPa resulted in a significant decrease in the intrinsic viscosity and hydrodynamic radius of pepsin (*p* < 0.05) and an obvious increase in the surface hydrophobicity (*p* < 0.05). At pressures higher than 100 MPa, both the intrinsic viscosity and hydrodynamic radius values rose, while the surface hydrophobicity decreased. Extended treatment times at a pressure of 100 MPa could lead to similar changing trends. It could be inferred that the high-pressure treatment was able to improve the hydration of pepsin, resulting in a decrease of intrinsic viscosity and hydrodynamic radius of pepsin. Additionally, during the high-pressure treatment, some noncovalent bonds were destroyed, resulting in a more exposed hydrophobic region. High-pressure treatment with pressure higher than 150 MPa and holding time more than 15 min could damage the interaction between pepsin and water.

### 2.5. Effect of High-Pressure Treatment on Subunit Composition of Pepsin

**Figure 4:**
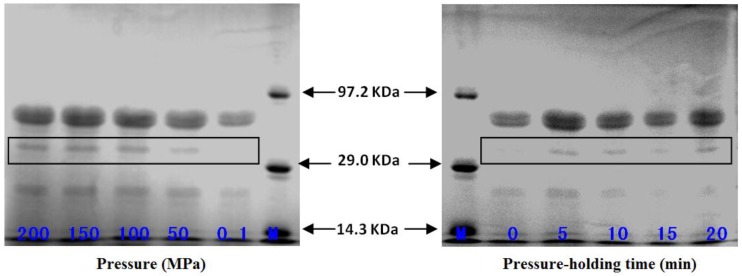
SDS-PAGE of pepsin treated under different pressures and pressure holding times.

The electrophoresed bands corresponding to the standard pepsin samples treated at pressures of 0.1, 50, 100, 150 and 200 MPa and pepsin samples treated at 100 MPa for 10, 15 and 20 min are shown in Figure 4. All the pepsin subunit bands can be clearly observed. The subunits of the pepsin with molecular weights of 37.0 kDa, 21.0 kDa and 15.0 kDa were consistent with the report by Samloff et al. [13], which demonstrates that the gastric mucosae of animals and humans are able to secrete a variety of pepsins (Pg 1–7) according to their different electrophoretic mobilities. Further, each zymogen is able to become an active enzyme after a certain number of peptide fragments had been removed. In another study, Castillo-Yanez et al., isolated and purified two acidic proteases with different molecular weights from the stomach of sardines [14]. As shown in Figure 4, the 37.0 kDa subunit of high pressure-treated pepsin became thicker compared with the control group (0.1 MPa), but there were no significant changes in this subunit band among the pressure treatment groups (50, 100 and 200 MPa). When the reactions were incubated at a pressure of 100 MPa for different pressure holding times, the 37.0 kDa subunit band changed significantly compared with the control group (0 min). This suggested that high-pressure treatment allows mutual conversion among the 37.0 kDa, 21.0 kDa and 15.0 kDa subunits of pepsin, which improves the enzyme activity to varying degrees.

## 3. Experimental Section

### 3.1. Chemicals

Pepsin was purchased from Jingchun Biochemical Technology Co., Ltd. (Shanghai, China). Casein was obtained from Beijing Solarbio Technology Co., Ltd., Beijing, China, and bovine serum albumin and L-tyrosine were purchased from Beijing Aobox Biotechnology Co., Ltd., Beijing, China. Other chemical reagents used were of analytical grade. 

### 3.2. High-Pressure Treatment of Pepsin

A specific amount of pepsin was dissolved in hydrochloric acid solution (pH 1.5) to a concentration of 2 mg/mL. Then, the obtained enzyme solution was vacuum-packed and placed in a UHPF-750 MPa ultra-high pressure processing apparatus (Baotou Kefa New High-tech Food Machinery Co., Ltd., Baotou, China). The high-pressure treatment was performed at 25 °C under different pressures (50, 100, 150 and 200 MPa) for different durations (5, 10, 15 and 20 min). The enzyme solution that did not undergo high-pressure treatment (atmospheric pressure, 0.1 MPa) was used as a control sample.

### 3.3. Measurement of Enzymatic Activity of Pepsin

The enzymatic activity of pepsin was measured by using the Folin-Ciocalteu method according to a previous report [15]. One enzyme activity unit (U) is defined as the amount of enzyme that is required to release 1 μg of tyrosine from the hydrolysis of 1 g of casein per minute. 

### 3.4. Determination of Degree of Hydrolysis

The degree of hydrolysis of casein was determined by using the formaldehyde titration method [16]. Briefly, 5 mL of the casein hydrolysate was diluted to 100 mL. After being mixed evenly, 20 mL of the obtained solution was placed in a 200 mL beaker, 60 mL of water was added, and the solution was titrated to pH 8.2 with 0.05 mol/L standard NaOH solution. Then, 10 mL of formaldehyde solution was added, and the solution was mixed evenly and further titrated to pH 9.2 with 0.05 mol/L NaOH. Distilled water was used instead of hydrolysate as the blank control. The volume of standard NaOH solution consumed was recorded to calculate the degree of hydrolysis (DH) as follows:

(1) $$DH(\%)=\frac{h}{h_{tot}}\;h=\frac{C\times (V_{1}-V_{2})}{V_{3}\cdot S\cdot W}$$

where C is the concentration of the standard NaOH solution (mol/L), V_1_ is the volume of sodium hydroxide standard consumed in sample titration (mL); V_2_ is the volume of sodium hydroxide standard consumed in the blank control titration (mL), V_3_ is the volume of the diluted reagent, S is the substrate concentration, W is the protein content of the casein sample used (87.34% in this experiment), h_tot_ is the total number of peptide bonds in the protein (mmol/g; 8.2 × 10^−3^ mmol/g for casein).

### 3.5. Determination of Molecular Weight Distribution of Casein Hydrolysate

Casein was added gradually to the high pressure-treated enzyme solution until the casein concentration reached 10 mg/mL, and the mixture underwent enzymatic hydrolysis in a water bath oscillator at 30 °C for 4 h. After hydrolysis, the enzyme was denatured in a boiling water bath for 10 min, and the reaction solution was cooled to room temperature and stored at 4 °C until analysis. The molecular weight distribution of peptides of the hydrolysate was determined by using a Waters 600 high-performance liquid chromatography (Milford, MA, USA) coupled with a 2487 ultraviolet detector, an Empower workstation and gel permeation chromatography (GPC) software. The sample was separated on a TSKgel 2000 SWXL separation column (300 × 7.8 mm). The mobile phase consisted of acetonitrile/water/trifluoroacetic acid (45/55/0.1). The flow rate was set at 0.5 mL/min and the column temperature was at 30 °C. The peptide standards included cytochrome C (MW 12,384), bacitracin (MW 1450), Gly-Gly-Tyr-Arg (MW 451), and Gly-Gly-Gly (MW 189). The injection volume was 10 μL. The detection wavelength was 220 nm.

### 3.6. Determination of the Sulfhydryl Group (SH) and Disulfide Bond (S-S) Contents of Pepsin

The SH and S-S contents of high pressure-treated pepsin were measured by using the reported method by Beveridge et al. [17]. To measure the sulfhydryl group content, 2.5 mL of Tris-Gly-8 M urea followed by 0.02 mL of DTNB (Ellman’s reagent; 4 mg/mL) were added to 0.5 mL of high pressure-treated enzyme solution. After rapid mixing, the reaction was allowed to proceed at 25 °C for 30 min, and the absorbance at λ = 412 nm (A_412_) was measured using a TU-1810 UV spectrophotometer (Purkinje, Beijing, China). A blank (distilled water) was measured at the same time. The SH content (A1) was calculated as follows:

A1 = 73.53 × A_412_ × D/C (2)

where A_412_ is the absorbance value at λ = 412 nm; D is the dilution factor (6.04 in the current experiment); and C is the final protein concentration in mg/mL.

To measure the S-S content, 0.2 mL of enzyme solution and 1.0 mL of Tris-Gly-10 M urea were mixed and allowed to stand at 25 °C for 1 h before 10 mL of 12% trichloroacetic acid (TCA) was added. The mixture was allowed to stand at the same temperature for another hour and was centrifuged at 3000 rpm for 10 min. The precipitate was then washed with 12% TCA and centrifuged; this was performed twice. Finally, 3 mL of Tris-Gly-8 M urea and 0.03 mL of DTNB were added to the centrifuged residue. After rapid mixing, the reaction at was allowed to proceed at 25 °C for 30 min, and the absorbance at λ = 412 nm (A_412_) was read using a TU-1810 UV spectrophotometer (Purkinje, Beijing, China). A blank sample (distilled water) was measured at the same time. The S-S content was calculated as follows:

A2 = 73.53 × A_412_ × D/C (3)

where D = 15, and the remaining values are the same as those described above in the formula to calculate the SH bond content.

### 3.7. Determination of Surface Hydrophobicity Index of Pepsin

The ANS (1-anilino-8-naphthalene sulfonate) fluorescence probe was used to measure the surface hydrophobicity index of high pressure-treated enzyme [18]. The protease concentration in high pressure-treated enzyme solution that had been serially diluted in phosphate buffer (concentration range, 0.005–0.1%) was measured using the Kjeldahl method. Briefly, 5 mL of sample solutions with different concentrations and 50 μL of 8 mmol/L ANS (prepared using 0.01 mol/L phosphate buffer, pH = 7.0) were combined and mixed by shaking. The fluorescence intensity (FI) was measured after the mixture was allowed to stand for 3 min at the excitation wavelength (λ_ex_) of 338 nm and the emission wavelength (λ_em_) of 496 nm. The fluorescence intensity was plotted against the protein concentration, and the slope of the initial section was the surface hydrophobicity index (So) of the protein.

### 3.8. Determination of Intrinsic Viscosity and Hydrodynamic Radius of Pepsin

The effect of high-pressure treatment on the intrinsic viscosity and hydrodynamic radius of pepsin was determined by using a Viscotek 270 max high performance size exclusion chromatography (Malvern, UK), which included the following detectors: refractive index, viscosity and dual-angle laser-light scattering. The sample was separated on a Viscotek P3000 column (300 × 8.0 mm) at 30 °C with bovine serum albumin as standard. The mobile phase consisted of 0.25 mol/L NaCl in phosphate buffer (pH 7.2) using a flow rate of 0.5 mL/min and an injection volume of 100 μL. The data was recorded and processed by Omnisec V5.0 software (Malvern, UK). 

### 3.9. Determination of Subunit Composition of Pepsin

The subunit composition was determined by using a published method [19]. The sample was separated using discontinuous sodium dodecyl sulfate-polyacrylamide gel electrophoresis (SDS-PAGE) with a 5% stacking gel and a 10% separating gel at 70 V, and low molecular weight standard proteins (14.4–97.4 KDa) were used. After electrophoresis, the gel was fixed, stained, and destained, and the images were captured on film using the gel imaging and analysis system. The differences in subunits among the treatment groups were analyzed.

### 3.10. Statistical Analysis

The data obtained were expressed as the mean of three replicate determinations plus or minus the standard deviation (SD). Statistical comparisons were made with Student’s test. *p* values < 0.05 were considered to be significant.

## 4. Conclusions

In conclusions, the high-pressure treatment could lead to changes in sulfhydryl group/disulfide bond content, hydrophobicity, hydrodynamic radius, intrinsic viscosity, and subunit composition of pepsin, and the conformational change of pepsin resulted in the improvement of its enzymatic activity and the efficiency of hydrolysis, which had an obvious relationship with the treatment conditions. The obtained results may lay the foundation for further exploration of the mechanisms underlying the ability of high-pressure treatment to promote enzymatic hydrolysis of proteases.

## Figures and Tables

**Table 1:** Effect of the pressure on the molecular weight distribution of casein hydrolysate.

Pressure (MPa)	Molecular Weight Distribution (%)				
	>3000	3000–2000	2000–1000	1000–500	<500
0	7.82	9.5	20.61	17.65	44.42
50	6.25	8.79	21.35	17.55	46.06
100	6.22	8.66	21.48	17.52	46.39
150	6.24	8.77	21.11	17.58	46.31
200	6.43	8.85	20.94	17.38	46.11

**Table 2:** Effect of the pressure-holding time on the molecular weight distribution of casein hydrolysate.

Pressure Holding Time (min)	Molecular Weight Distribution (%)				
	>3000	3000–2000	2000–1000	1000–500	<500
0	8.3	9.39	20.8	17.59	43.91
5	6.98	8.75	21	17.57	45.64
10	6.96	8.71	21.26	17.41	45.73
15	6.48	8.7	21.41	17.4	45.93
20	6.91	8.79	21.18	17.4	45.85
